# Supplementary material for: Pyruvate Dehydrogenase Kinase 1 inhibition mediated oxidative phosphorylation enhancement in cartilage promotes osteoarthritis progression
Source: BMC Musculoskelet Disord. 2023 Jul 20;24:597. doi: 10.1186/s12891-023-06585-6 (PMC10357736; doi:10.1186/s12891-023-06585-6)
Supplement: Supplementary file 1 — Additional file 1. [file 12891_2023_6585_MOESM1_ESM.zip › MF.Gsea.1653627316242/GOMF_COPPER_ION_BINDING.html]

Details for gene set GOMF\_COPPER\_ION\_BINDING[GSEA]

|  || Dataset | 55457\_collapsed\_to\_symbols.cls.cls #OA\_versus\_normal.cls.cls #OA\_versus\_normal\_repos |
| Phenotype | cls.cls#OA\_versus\_normal\_repos |
| Upregulated in class | OA |
| GeneSet | GOMF\_COPPER\_ION\_BINDING |
| Enrichment Score (ES) | 0.32557362 |
| Normalized Enrichment Score (NES) | 1.2992936 |
| Nominal p-value | 0.11508951 |
| FDR q-value | 0.4068108 |
| FWER p-Value | 1.0 |
Table: GSEA Results Summary

  

Fig 1: Enrichment plot: GOMF\_COPPER\_ION\_BINDING      
 Profile of the Running ES Score & Positions of GeneSet Members on the Rank Ordered List

  

| SYMBOL | TITLE | RANK IN GENE LIST | RANK METRIC SCORE | RUNNING ES | CORE ENRICHMENT || 1 | COX17 | cytochrome c oxidase copper chaperone COX17 [Source:HGNC Symbol;Acc:HGNC:2264] | 64 | 0.966 | 0.0596 | Yes |
| 2 | SOD3 | superoxide dismutase 3 [Source:HGNC Symbol;Acc:HGNC:11181] | 608 | 0.620 | 0.0589 | Yes |
| 3 | LOX | lysyl oxidase [Source:HGNC Symbol;Acc:HGNC:6664] | 872 | 0.550 | 0.0752 | Yes |
| 4 | F8 | coagulation factor VIII [Source:HGNC Symbol;Acc:HGNC:3546] | 1292 | 0.459 | 0.0733 | Yes |
| 5 | PARK7 | Parkinsonism associated deglycase [Source:HGNC Symbol;Acc:HGNC:16369] | 1406 | 0.442 | 0.0941 | Yes |
| 6 | CUTA | cutA divalent cation tolerance homolog [Source:HGNC Symbol;Acc:HGNC:21101] | 1415 | 0.441 | 0.1230 | Yes |
| 7 | ADNP | activity dependent neuroprotector homeobox [Source:HGNC Symbol;Acc:HGNC:15766] | 1466 | 0.434 | 0.1481 | Yes |
| 8 | LOXL1 | lysyl oxidase like 1 [Source:HGNC Symbol;Acc:HGNC:6665] | 1494 | 0.429 | 0.1748 | Yes |
| 9 | TYR | tyrosinase [Source:HGNC Symbol;Acc:HGNC:12442] | 1547 | 0.421 | 0.1989 | Yes |
| 10 | COX11 | cytochrome c oxidase copper chaperone COX11 [Source:HGNC Symbol;Acc:HGNC:2261] | 1622 | 0.410 | 0.2206 | Yes |
| 11 | SNCA | synuclein alpha [Source:HGNC Symbol;Acc:HGNC:11138] | 1800 | 0.384 | 0.2325 | Yes |
| 12 | P2RX4 | purinergic receptor P2X 4 [Source:HGNC Symbol;Acc:HGNC:8535] | 1827 | 0.381 | 0.2559 | Yes |
| 13 | CUTC | cutC copper transporter [Source:HGNC Symbol;Acc:HGNC:24271] | 1978 | 0.363 | 0.2685 | Yes |
| 14 | AOC1 | amine oxidase copper containing 1 [Source:HGNC Symbol;Acc:HGNC:80] | 1992 | 0.362 | 0.2917 | Yes |
| 15 | ANG | angiogenin [Source:HGNC Symbol;Acc:HGNC:483] | 2009 | 0.360 | 0.3146 | Yes |
| 16 | S100A13 | S100 calcium binding protein A13 [Source:HGNC Symbol;Acc:HGNC:10490] | 2358 | 0.319 | 0.3088 | Yes |
| 17 | SOD1 | superoxide dismutase 1 [Source:HGNC Symbol;Acc:HGNC:11179] | 2413 | 0.313 | 0.3256 | Yes |
| 18 | CCS | copper chaperone for superoxide dismutase [Source:HGNC Symbol;Acc:HGNC:1613] | 2727 | 0.278 | 0.3198 | No |
| 19 | RNF7 | ring finger protein 7 [Source:HGNC Symbol;Acc:HGNC:10070] | 3264 | 0.225 | 0.2931 | No |
| 20 | ATOX1 | antioxidant 1 copper chaperone [Source:HGNC Symbol;Acc:HGNC:798] | 3290 | 0.221 | 0.3060 | No |
| 21 | SNCG | synuclein gamma [Source:HGNC Symbol;Acc:HGNC:11141] | 4042 | 0.152 | 0.2577 | No |
| 22 | HEPH | hephaestin [Source:HGNC Symbol;Acc:HGNC:4866] | 4170 | 0.140 | 0.2572 | No |
| 23 | F5 | coagulation factor V [Source:HGNC Symbol;Acc:HGNC:3542] | 4223 | 0.135 | 0.2622 | No |
| 24 | PAM | peptidylglycine alpha-amidating monooxygenase [Source:HGNC Symbol;Acc:HGNC:8596] | 4373 | 0.123 | 0.2588 | No |
| 25 | ATP7A | ATPase copper transporting alpha [Source:HGNC Symbol;Acc:HGNC:869] | 4814 | 0.086 | 0.2304 | No |
| 26 | CP | ceruloplasmin [Source:HGNC Symbol;Acc:HGNC:2295] | 4833 | 0.085 | 0.2346 | No |
| 27 | TP53 | tumor protein p53 [Source:HGNC Symbol;Acc:HGNC:11998] | 5511 | 0.032 | 0.1841 | No |
| 28 | ATP7B | ATPase copper transporting beta [Source:HGNC Symbol;Acc:HGNC:870] | 5751 | 0.012 | 0.1663 | No |
| 29 | DBH | dopamine beta-hydroxylase [Source:HGNC Symbol;Acc:HGNC:2689] | 6133 | -0.015 | 0.1376 | No |
| 30 | ATP13A2 | ATPase cation transporting 13A2 [Source:HGNC Symbol;Acc:HGNC:30213] | 6336 | -0.031 | 0.1240 | No |
| 31 | MOXD1 | monooxygenase DBH like 1 [Source:HGNC Symbol;Acc:HGNC:21063] | 6813 | -0.064 | 0.0913 | No |
| 32 | APOA4 | apolipoprotein A4 [Source:HGNC Symbol;Acc:HGNC:602] | 6817 | -0.064 | 0.0953 | No |
| 33 | LOXL2 | lysyl oxidase like 2 [Source:HGNC Symbol;Acc:HGNC:6666] | 7675 | -0.133 | 0.0375 | No |
| 34 | ALB | albumin [Source:HGNC Symbol;Acc:HGNC:399] | 7684 | -0.133 | 0.0458 | No |
| 35 | IL1A | interleukin 1 alpha [Source:HGNC Symbol;Acc:HGNC:5991] | 7842 | -0.144 | 0.0432 | No |
| 36 | PRNP | prion protein [Source:HGNC Symbol;Acc:HGNC:9449] | 9042 | -0.235 | -0.0344 | No |
| 37 | AOC3 | amine oxidase copper containing 3 [Source:HGNC Symbol;Acc:HGNC:550] | 9375 | -0.262 | -0.0427 | No |
| 38 | S100A12 | S100 calcium binding protein A12 [Source:HGNC Symbol;Acc:HGNC:10489] | 10113 | -0.332 | -0.0778 | No |
| 39 | PRND | prion like protein doppel [Source:HGNC Symbol;Acc:HGNC:15748] | 10541 | -0.377 | -0.0858 | No |
| 40 | DCT | dopachrome tautomerase [Source:HGNC Symbol;Acc:HGNC:2709] | 10995 | -0.429 | -0.0924 | No |
| 41 | S100A5 | S100 calcium binding protein A5 [Source:HGNC Symbol;Acc:HGNC:10495] | 10997 | -0.429 | -0.0637 | No |
| 42 | GPC1 | glypican 1 [Source:HGNC Symbol;Acc:HGNC:4449] | 11096 | -0.443 | -0.0417 | No |
| 43 | SCO2 | synthesis of cytochrome C oxidase 2 [Source:HGNC Symbol;Acc:HGNC:10604] | 11410 | -0.488 | -0.0334 | No |
| 44 | AOC2 | amine oxidase copper containing 2 [Source:HGNC Symbol;Acc:HGNC:549] | 11550 | -0.508 | -0.0103 | No |
| 45 | ACR | acrosin [Source:HGNC Symbol;Acc:HGNC:126] | 11880 | -0.567 | 0.0020 | No |
| 46 | HAMP | hepcidin antimicrobial peptide [Source:HGNC Symbol;Acc:HGNC:15598] | 11909 | -0.573 | 0.0382 | No |
| 47 | SNCB | synuclein beta [Source:HGNC Symbol;Acc:HGNC:11140] | 11941 | -0.580 | 0.0746 | No |
Table: GSEA details [plain text format]

  

Fig 2: GOMF\_COPPER\_ION\_BINDING      
 Blue-Pink O' Gram in the Space of the Analyzed GeneSet

  

Fig 3: GOMF\_COPPER\_ION\_BINDING: Random ES distribution      
 Gene set null distribution of ES for **GOMF\_COPPER\_ION\_BINDING**
